# T2 quantification for improved detection of myocardial edema

**DOI:** 10.1186/1532-429X-11-56

**Published:** 2009-12-30

**Authors:** Shivraman Giri, Yiu-Cho Chung, Ali Merchant, Georgeta Mihai, Sanjay Rajagopalan, Subha V Raman, Orlando P Simonetti

**Affiliations:** 1 Department of Biomedical Engineering, The Ohio State University, Columbus, Ohio, USA; 2 Dorothy M Davis Heart & Lung Research Institute, The Ohio State University, Columbus, Ohio, USA; 3 Department of Internal Medicine, Division of Cardiovascular Medicine, The Ohio State University, Columbus, Ohio, USA; 4 Siemens Healthcare, USA; 5 Department of Radiology, The Ohio State University, Columbus, Ohio, USA

## Abstract

**Background:**

T2-Weighted (T2W) magnetic resonance imaging (MRI) pulse sequences have been used to detect edema in patients with acute myocardial infarction and differentiate acute from chronic infarction. T2W sequences have suffered from several problems including (i) signal intensity variability caused by phased array coils, (ii) high signal from slow moving ventricular chamber blood that can mimic and mask elevated T2 in sub-endocardial myocardium, (iii) motion artifacts, and (iv) the subjective nature of T2W image interpretation. In this work we demonstrate the advantages of a quantitative T2 mapping technique to accurately and reliably detect regions of edematous myocardial tissue without the limitations of qualitative T2W imaging.

**Methods:**

Methods of T2 mapping were evaluated on phantoms; the best of these protocols was then optimized for *in vivo *imaging. The optimized protocol was used to study the spatial, view-dependent, and inter-subject variability and motion sensitivity in healthy subjects. Using the insights gained from this, the utility of T2 mapping was demonstrated in a porcine model of acute myocardial infarction (AMI) and in three patients with AMI.

**Results:**

T2-prepared SSFP demonstrated greater accuracy in estimating the T2 of phantoms than multi-echo turbo spin echo. The T2 of human myocardium was found to be 52.18 ± 3.4 ms (range: 48.96 ms to 55.67 ms), with variability between subjects unrelated to heart rate. Unlike T2W images, T2 maps did not show any signal variation due to the variable sensitivity of phased array coils and were insensitive to cardiac motion. In the three pigs and three patients with AMI, the T2 of the infarcted region was significantly higher than that of remote myocardium.

**Conclusion:**

Quantitative T2 mapping addresses the well-known problems associated with T2W imaging of the heart and offers the potential for increased accuracy in the detection of myocardial edema.

## Background

Many pathologic conditions alter the myocardial tissue water content, which in turn alters the T2 relaxation time. Higgins et al. [1] showed in 1983 a direct relation between T2 relaxation time and myocardial water content in a canine model of acute myocardial infarction (AMI). More recently, studies on animals [2-4] and patients [5-7] have demonstrated that increased myocardial T2 may be associated not only with AMI, but also with severe transient myocardial ischemia. Elevated myocardial T2 is also known to accompany myocarditis [8] and cardiac allograft rejection [9]. These and other studies using T2 to characterize pathological changes in myocardium have relied on T2-Weighted (T2W) imaging with a dark blood turbo spin echo technique [10]. Recent review [11] and editorials [12,13] have acknowledged a set of challenges that continue to limit the widespread clinical acceptance of T2W imaging. These limitations include: i) phased-array coils cause regional myocardial signal variation that can lead to inaccurate interpretation and diagnosis; ii) high signal from stagnant blood makes it difficult to differentiate edema from sub-endocardial blood; iii) myocardial signal loss caused by through-plane motion; and iv) the qualitative nature of T2W imaging where interpretation depends on regional differences in myocardial signal intensity, which may vary depending on sequence parameters (echo times, slice thickness etc.). Two recently introduced techniques [14,15] address several of these listed deficiencies, but these new methods are also qualitative and still depend on subjective interpretation of T2W images and relative regional differences in myocardial signal intensity.

An alternative approach to T2W imaging is to directly quantify the T2 of the myocardium. Using quantitative T2-mapping, the artifacts associated with T2W imaging may be minimized, image contrast dependency on user-defined parameters and subjective interpretation can be reduced, and subtle T2 differences between tissues may be more easily detected. Over the past 25 years a number of *in vivo *studies have measured the T2 of healthy human myocardium (Table 1), primarily using techniques based on spin echo acquisitions. These methods are time-consuming and prone to the same motion sensitivity that has plagued T2W imaging of the heart. Myocardial T2-mapping techniques using T2-prepared steady-state free precession (T2p-SSFP) have been described recently by Huang et al. [16] for BOLD imaging, and as a combined T1 and T2 mapping method by Blume *et al*. [17] for differentiation of acute from chronic myocardial infarction. While these were promising initial studies, neither investigated the potential for T2-mapping to address the known limitations of T2W imaging caused by residual blood signal, surface coil intensity variation, and myocardial motion artifact.

**Table 1:** Previously published *in-vivo *human myocardial T2 values.

Year	T2 (ms)	Authors	Reference
1984	57 ± 16	Bottomley et al.	Med Phys. 1984 Jul-Aug;11(4):425-48.
1985	42.4^1^	McNamara et al.	Circulation. 1985:71, No. 4, 717-724.
1993	50 ± 3	Walker et al.	Magn Reson Med. 1993 May;29(5):637-41.
1998	44.3 ± 3.5	Mavrogeni et al.	MAGMA. 1998 Aug;6(1):7-12
1998	48.3 ± 5.5	Mavrogeni et al.	Int J Card Imaging. 1998 Apr;14(2):117-22
2000	57.95 ± 4.9	Papanikolaou et al.	Acta Radiol. 2000 Jul;41(4):348-51.
2003	52 ± 4.3	Foltz et al.	Magn Reson Med. 2003 Jun;49(6):1089-97.
2005	58.1 ± 7.1	Mavrogeni et al.	Chest. 2005 Jan;127(1):143-8.
2006	56.9 ± 8.4	He et al.	J Magn Reson Imaging. 2006 Sep;24(3):580-5
2007	54 ± 4.3	Huang et al.	Magn Reson Med. 2007 May;57(5):960-6.
2009	50 ± 4	Blume et al.	J Magn Reson Imaging. 2009 Feb;29(2):480-7
2009	54 ± 6.8	Sparrow et al.	J Magn Reson Imaging. 2009 Nov;30(5):942-6.

^1 ^Indicates the T2 measured in the myocardium of normal volunteers, as reported by the study. The paper also reported T2 of normal (remote) myocardium in patients; the value was 42.3 ms.

The objectives of the present work were to i) demonstrate a practical T2-mapping method based on T2p-SSFP that minimizes the problems of current T2W imaging methods; ii) report a comprehensive analysis of T2 computed over the entire myocardium vis-à-vis variation across slice positions and orientations, subjects, and myocardial segments; iii) demonstrate this technique in a porcine model of AMI, and in patients with AMI. We hypothesize that quantitative T2-mapping addresses the limitations of qualitative T2W imaging and provides a practical and accurate method of assessing myocardial edema associated with acute ischemia, infarction, and other pathological conditions affecting myocardial water content.

## Materials and methods

The T2-mapping pulse sequence was developed on a 1.5 T MR system (Avanto, Siemens Healthcare, Erlangen, Germany); the T2-map calculation algorithm was implemented in MATLAB (The Mathworks Inc., Natick, MA, USA.). In all hypotheses testing, a p-value < 0.05 was considered statistically significant. All analyses were performed using MINITAB statistical software package (Minitab Inc., State College PA, USA.).

### T2-prepared SSFP pulse sequence

A T2-prepared steady-state free precession sequence [15] was used to generate three T2W images, one each with different T2 preparation times (TE_T2P _= 0 ms, 24 ms, 55 ms). Table 2 lists the imaging parameters. Because these images are acquired in the transient state of single-shot SSFP immediately after the T2 preparation pulse, the primary source of contrast is the T2 relaxation time. Thus, the signal in each image represents a different echo time along the T2 decay curve.

**Table 2:** Imaging parameters

Parameter	T2 Mapping		T2W
		
	T2p-SSFP	MESE	
Echo time (ms)	0, 24, 55	[4.4, 53], [4.4, 44, 84]	62
TR	3 × RR	3 × RR	2 × RR
Image matrix	96 × 160	85 × 128	108 × 192
Echo spacing (ms)	2.6	4.4	5.2
Acquisition	Single shot	segmented	segmented
ETL per image	NA	11, 9	29
Acquisition time	7 × RR	18 × RR, 21 × RR	10 × RR
Flip angle	40°	90°	90°
Bandwidth (Hz/pixel)	1488	482	347
Parallel acquisition (Technique/Acceleration)	GRAPPA/2	GRAPPA/2	GRAPPA/2

**MESE **Multi Echo Spin Echo; **T2p-SSFP **T2-Prepared Steady State Free Precession; **T2W **T2-Weighted; **GRAPPA **Generalized autocalibrating partially parallel acquisition.

The T2-prepared single-shot SSFP sequence was similar to that described by Huang et al. [16]. The T2 preparation scheme (T2p) was identical to that described in an earlier work [18]. The design incorporates non-selective composite pulses for insensitivity to motion and B0 and B1 inhomogeneities. The SSFP readout module was applied immediately after the T2p to sample the magnetization prior to reaching the SSFP steady state. Prior work [19,20] has suggested steady-state preparation schemes to reduce the oscillations that occur in the transient state of SSFP due to off-resonance, and the linear flip angle (LFA) approach was shown to have a superior performance in the presence of large off-resonance frequencies [19]. We thus chose the LFA scheme with 10 steps to stabilize the transient oscillations. Both linear and centric ordered acquisitions of k-space data were evaluated.

**Figure 1:**
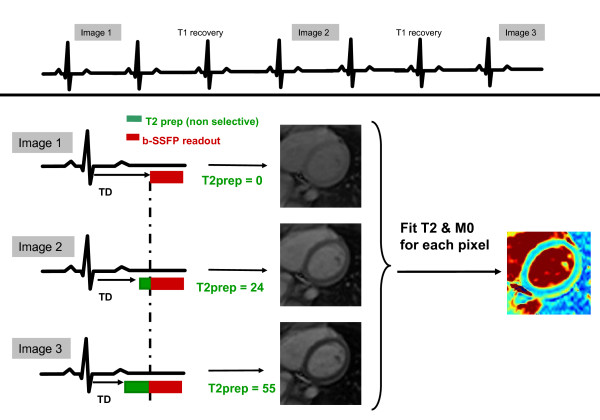
**T2 mapping scheme**. Three images were acquired with different T2 preparation times with a gap of 2 RR intervals to allow for sufficient T1 recovery. Seven heartbeats were required for image acquisition performed during breath-hold. Trigger delay (TD) was adjusted for each of the three images to ensure that the readout was always in the same phase of cardiac cycle. The three acquired images were processed to fit the T2 decay curve at each pixel to yield a T2 map.

T2 maps were generated by fitting the following two-parameter equation to corresponding pixels from each of the three images:

where *S(x, y) *is signal intensity, *M*_0_*(x, y) *is a lumped parameter that includes the equilibrium magnetization and local receiver coil gain, and TE_T2P _is the T2 preparation time. The above equation was fit using a linear 2-parameter model after logarithmic transformation. To minimize breath-hold duration (and thus patient discomfort), we limited the number of TE_T2P _times to three, the minimum required for a linear least-squares fit. A wait time of 2 RR intervals was inserted between images to allow for sufficient relaxation; thus, a total of 7 RR intervals were required to acquire the three T2p images. The sequence timing was adjusted for each T2p time to ensure that the SSFP readout was always timed to the same phase of the cardiac cycle. The entire scheme (image acquisition + T2 map generation) is depicted in Figure 1.

The TE_T2P _times (0 ms, 24 ms, and 55 ms) were chosen based on the expected range of T2 values in the myocardium. Recent work (see Table 1) has shown that the T2 for normal myocardium is approximately 55 ms; accordingly, we chose the longest TE_T2P _to be 55 ms because longer TE_T2P _would cause significant signal loss. The recent paper by Blume et al. [17] showed that myocardial T2 estimation was improved by including an image without T2 preparation (TE_T2P _= 0 ms). TE_T2P _= 24 ms was the minimum time permitted by the preparation scheme and was chosen as an intermediate point on the curve to support a least-squares fit.

### Phantom study

Phantom studies were performed to evaluate the accuracy of the T2p-SSFP method. Phantoms were prepared using two different mixtures of NiCl_2 _and agar [21] to approximate the T1 and T2 values of normal (T1/T2 = 1081/51 ms) and edematous (T1/T2 = 1223/62 ms) myocardium [14]. The T1 values were verified using an inversion recovery spin echo sequence with eighteen TI times ranging from 22 to 3000 ms; the T2 values were verified using a spin echo sequence with thirteen TE times ranging from 11 to 143 ms. Both spin echo protocols used a TR of 10 seconds to allow for complete recovery of longitudinal magnetization and the scan time per image was approximately 17 minutes. Both T1 and T2 were fit using Levenberg-Marquardt non-linear least squares method. T2p-SSFP was run using both linear and centric k-space ordering to evaluate the performance of each. All phantom studies were performed with a simulated heart rate of 60 beats per min (bpm). Different wait times (2 through 5 RR) between TE_T2P _images were compared to evaluate the influence of T1 recovery on the accuracy of the T2 maps. The phantom T2's were also measured using a conventional multi-echo turbo spin echo (MESE) approach to compare it's accuracy with T2p-SSFP. Two MESE protocols were used: 2 echoes (TE = 4.4, 53 ms) and 3 echoes (TE = 4.4, 44, 84 ms) (see Table 2 for imaging parameters).

### In-vivo Measurements

With the insights gained from phantom studies, six healthy volunteers were studied first to evaluate the differences between linear and centric ordering of the T2p-SSFP acquisition. Thereafter, the chosen k-space ordering scheme was used to investigate the *in-vivo *T2 of human myocardium in seventeen healthy subjects. Three pigs with verified acute myocardial infarction and three patients, also with acute myocardial infarction, were scanned to investigate the ability of the T2p-SSFP technique to detect myocardial edema. All twenty three healthy subjects (18-33 years old) and three patients (ages: 55, 66, 79 years) gave written informed consent to participate in this HIPAA-compliant study approved by the institution's Human Subjects Committee; the animal study was approved by the Institutional Laboratory Animal Care and Use Committee.

### Healthy Subjects

Linear and centric k-space ordering were compared in six healthy subjects. A mid-ventricular short-axis (SAX) slice was scanned in each subject using both k-space orderings. The resulting mean T2 values and mean spatial T2 variation were compared. Coefficient of variation was used as a measure of spatial variation.

In fourteen additional normal subjects the following slices were imaged: 3 SAX at basal, mid and apical levels of left ventricle, horizontal long axis (HLA) and vertical long axis (VLA). The imaging parameters are described in Table 2; linear ordering of k-space was used. The images were segmented into 16 standard myocardial sectors [22] (6 basal sectors, 6 mid-ventricular, and 4 apical) for quantitative T2 analysis. Apical sectors from HLA and VLA views were used in four cases in which partial volume effects made it difficult to mark the myocardial borders in the apical SAX slice.

Using these data (n = 224 segments, 16 for each of the 14 subjects), inter-subject and spatial variability were assessed as described in following paragraphs.

ANOVA was performed to test inter-subject T2 variability. To test for spatial variability of T2 in the short-axis images from the 14 subjects (16 segments per subject, total n = 224 segments), the data was first adjusted against inter-subject variability by subtracting the mean T2 of the subject from the segment T2 values; variance of T2 across subjects was assumed to be equal. ANOVA was performed to test the hypothesis of equal mean among the 16 segments. Coefficient of variation was used as a measure of variability. Additionally, a simple linear regression was performed to check for correlation between T2 and RR interval to investigate the influence of heart rate on T2 measurement. To test for differences in T2 calculated from short-axis or long-axis views, the four mid-ventricular segments that are represented in short-axis, HLA (inferoseptal and anterolateral), and VLA (anterior and inferior) views were used. Paired t-test was performed to assess differences. Additionally, to provide a measure of variability at pixel level, spatial variation of T2 was computed across all pixels in the mid-ventricular slice for each subject.

To study the effect of spatially-variant coil sensitivity on T2 maps, we separately acquired T2p-SSFP images for a single mid-ventricular SAX slice in each subject and generated T2 maps for comparison with T2W images acquired using the triple inversion recovery STIR sequence [10]. Only anterior coil elements were used to create a significant signal gradient from anterior to posterior myocardial regions. In each T2W image and T2 map, the six standard myocardial segments were evaluated for spatial variability after normalization by the segment with the minimum value. Coefficient of variation in these normalized segments was used as a measure of spatial variation.

To compare the motion sensitivity of T2W and T2 mapping techniques, a short axis slice was imaged close to the base in six of the subjects (three of the previous fourteen subjects + three new subjects). Two scans were run for each technique to acquire images at two cardiac phases: (i) 400 ms after the R-wave and (ii) mid-diastole; matched ROIs were drawn in the lateral wall of the four images thus acquired. Paired t-test was used to test the hypothesis of no difference in signal intensity (T2W) or T2 (T2 Map) between the images acquired at two different points in the cardiac cycle.

### Animals

Acute myocardial infarction was induced in three pigs to determine whether significant changes in measured T2 could be detected. The animals were anesthetized with ketamine (7.5 mg/lb) and acepromazine (0.25 mg/lb). A catheter was then guided under x-ray fluoroscopy via the femoral artery. Thereafter, a balloon occluder was guided through the catheter to the left anterior descending (LAD) coronary artery just distal to the first diagonal branch. Once in place, the occluder was inflated to produce total LAD occlusion to induce ischemia. The occlusion was maintained for 90 minutes to induce infarction following which the balloon was deflated. MRI was performed 3 days after reperfusion. During the scan, anesthesia was maintained using isoflurane in oxygen (1-3%) and mechanical ventilation (20 mmHg). ECG, blood pressure and pulse oximetry were monitored throughout the experimental period.

T2 maps were acquired in each animal in one mid-ventricular short-axis slice that included the affected LAD territory. This was followed by an injection of gadolinium-diethylenetriamine pentaacetic acid (Gd-DTPA, Magnevist, Bayer Health Care Pharmaceuticals) and Late Gadolinium Enhancement (LGE) imaging [23] to evaluate the extent of myocardial infarction. One-sided t-test was used to assess whether the T2 in the anterior segment (LAD territory) was significantly higher than that in the remote inferior segment.

### Patients

Three adult patients with acute myocardial infarction (AMI) were studied to gain insights into the potential clinical applications of this technique. Since the purpose of this study was only intended to evaluate the feasibility of myocardial T2 mapping, the inclusion criteria were restricted to patients with documented cases of AMI based on electrocardiography, x-ray coronary angiography, and cardiac biomarkers. Table 3 describes the clinical characteristic of each patient. MRI was performed on day 2 following the onset of symptoms. For edema imaging, both STIR and T2 maps were acquired. Edematous myocardial segments were defined from the results of coronary angiography and were compared with remote segments in both STIR images and T2 maps.

**Table 3:** Patient characteristics

Patient #	Age	Gender	ACS Type	Peak Troponin-I (ng/ml)	Coronary angiography
1	55	Male	STEMI	48	90% proximal RCA

2	79	Male	STEMI	47.8	100% proximal RCA

3	66	Male	STEMI	88.2	100% proximal LAD

**STEMI **ST segment Elevation Myocardial Infarction; **ACS **Acute Coronary Syndrome; **LAD **Left Anterior Descending; **RCA **Right Coronary Artery

## Results

### Phantom study

Tables 4 and 5 summarize the results from the phantom study. All sequences overestimated T2, although MESE resulted in the largest and T2p-SSFP with centric k-space ordering in the least overestimation (Table 4).

**Table 4:** Phantom study: comparison of MR pulse sequences.

		**T2 Map**			
			
	**True T2**	**T2p-SSFP**		**MESE**	
			
		**Centric**	**Linear**	**2 Echoes**	**3 Echoes**
	
Phantom 1	51 ± 0.6	53 ± 1.0	58 ± 1.0	64 ± 0.7	75 ± 2.6
Phantom 2	62 ± 1.1	63 ± 1.4	69 ± 1.4	80 ± 2	109 ± 16

Phantom T2 values (in ms) measured using T2p-SSFP (linear and centric k-space ordering) and MESE (two and three echoes). The true T2 values were measured using a spin-echo sequence with a TR of 10 seconds at the thirteen echo times listed in the text. A TR of 3 seconds was used for T2p-SSFP and MESE

Table 5 lists the results from the second phantom experiment to study the effect of the time interval between TE_T2P _image acquisitions.

**Table 5:** Phantom study: effect of wait times on T2.

	Wait times expressed as number (N) of RR intervals			
	N = 2	N = 3	N = 4	N = 5
**Phantom 1**	58 ± 1.0	59 ± 1.0	59 ± 1.1	60 ± 1.1
**Phantom 2**	69 ± 1.4	70 ± 1.5	71 ± 1.3	71 ± 1.4

Phantom T2 values (in ms) measured using T2p-SSFP sequence (linear k-space ordering) with different wait times (expressed in RR intervals) between TE_T2P _acquisitions

### Volunteer Results

**Figure 2:**
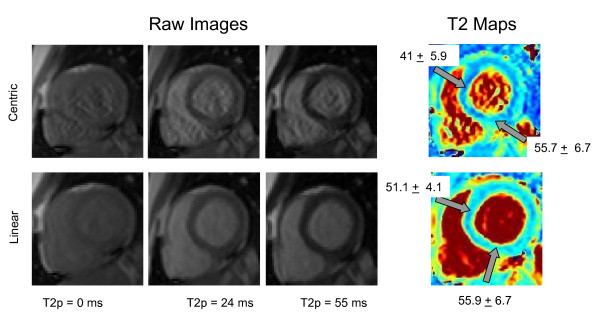
**Centric versus Linear k-space ordering in SSFP**. The raw images and T2 maps acquired with centric (top row) and linear (bottom row) ordering of k-space. Note the artifact in the raw images with centric ordering which is not present with linear ordering. The T2 maps generated using centric ordering showed greater variability than those acquired using linear k-space ordering.

Images acquired using centric reordering demonstrated artifacts in the blood pool and myocardium (Figure 2). These artifacts may be due to the initial transient oscillations caused by off-resonance frequencies affecting the central lines of k-space. Figure 2 shows images and T2 maps from one subject; the artifacts in centric reordered images caused regional heterogeneity in measured T2 values that could compromise diagnostic accuracy. Images acquired using linear ordering did not demonstrate such artifacts and the corresponding T2 maps were quite homogenous. The mean T2 values across these six subjects using the two methods were: 50.7 ± 2.4 ms for linear and 46.1 ± 3.5 ms for centric. The mean spatial variation with linear ordering was 4.2%, whereas that with centric ordering was 6.3%. In light of the better image quality and less variation of T2 maps, linear ordering was used for all subsequent studies in human subjects and in the animal model.

**Figure 3:**
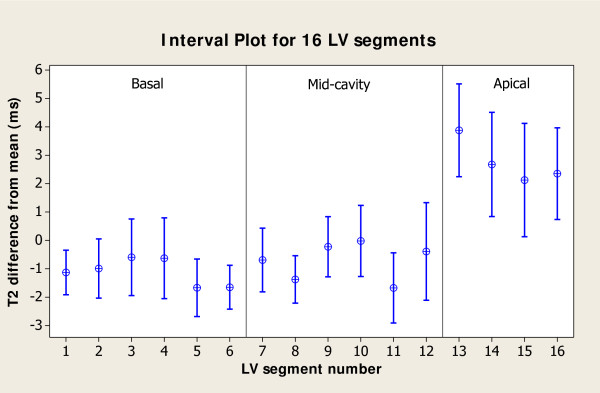
**Spatial variability in 16 LV myocardial segments**. The 95% CI of the adjusted T2 value (segment T2 - mean T2 in subject) across all subjects is plotted for each segment. The adjusted T2 values among LV segments reached statistical significance (p-value < 0.001) due to the higher apparent T2 measured in the apical slice.

The mean T2 value averaged across all 16 segments and among all 14 healthy subjects was found to be 52.18 ± 3.4 ms (range: 48.96 ms to 55.67 ms). ANOVA test for inter-subject variability of T2 values revealed statistically significant differences (p-value < 0.001). This variability showed no linear correlation with heart rate (R^2 ^= 0.018). The average coefficient of variation of T2 across subjects was 5.3%. Figure 3 shows the 95% CI for each of the 16 segments, suggesting that most of the variability was coming from the apical regions. ANOVA test for the basal and mid-ventricular segments showed no variation among these 12 segments results (p-value = 0.239). The mean spatial T2 variation for this subset of 12 segments was 3.8%. The T2 values computed from short-axis versus long-axis views in mid-ventricular segments did not show any significant variation (p-value = 0.319).

For the mid-ventricular SAX slice from fourteen subjects, the mean spatial variation expressed as coefficient of variation of the pixels was 8.7% ± 1.1%; the average number pixels per slice was 113.

**Figure 4:**
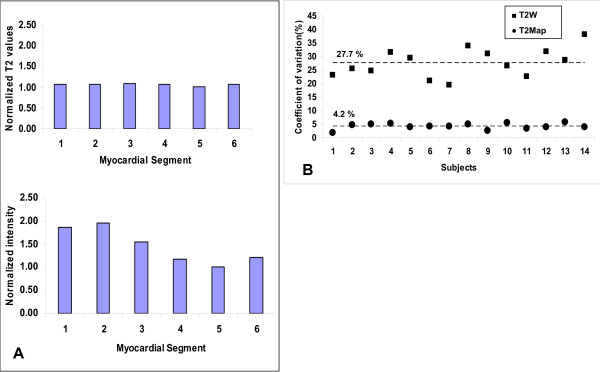
**Effect of surface phased array coils on T2 maps and T2W images; images acquired using anterior coils only**. **(A) **The graphs show normalized T2 values from T2 Maps (top) and normalized intensities from T2W images (bottom), averaged for all the 14 subjects. Myocardial Segments: 1 = Anterior, 2 = Anteroseptal, 3 = Inferoseptal, 4 = Inferior, 5 = Inferolateral, 6 = Anterolateral. **(B) **Spatial variability in T2W images (squares) and T2 Maps (dots) acquired using only anterior coil elements expressed as coefficient of variation. The mean spatial variation was 27.7% for T2W STIR and 4.25% for T2 Maps.

Figure 4A depicts the spatial variation in the 6 mid-ventricular myocardial segments for T2 maps and T2W images acquired in both cases using only anterior coil elements (averaged among 14 subjects). T2W images consistently showed higher signal for the anterior segment as compared to the inferior segment (p-value < 0.001; paired t-test). T2 maps did not show any such trend (p-value = 0.414; paired t-test), providing uniform signal in all myocardial segments. Figure 4-B summarizes the coefficient of variation for all the subjects. The mean coefficient of variation was 27.7% for T2W and 4.25% for T2 Maps.

**Figure 5:**
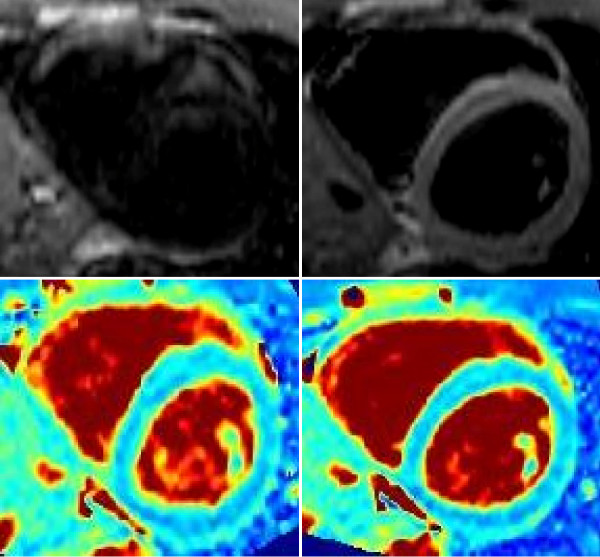
**Effect of cardiac motion on T2W images and T2 Maps**. Images were acquired 400 ms after R-wave (left column) and in mid-diastole (right column). T2W imaging (top row) showed sensitivity to cardiac through-plane motion with a near complete myocardial signal dropout. T2 map (bottom row) showed consistent results in both cardiac phases and demonstrated insensitivity to cardiac motion. Note that these images are from a more mobile basal slice and the images in left column were deliberately acquired in a highly dynamic cardiac phase to demonstrate insensitivity of T2 Maps to motion.

Figure 5 demonstrates the effect of cardiac motion on T2W and T2 Map. Note the near complete dropout of myocardial signal due to through-plane motion in T2W imaging when the image was acquired 400 ms after the R-wave; T2 Map, on the other hand, showed consistent results at each cardiac phase. In the six subjects, T2W imaging showed statistically significant difference in signal intensity (p-value = 0.002) in the lateral wall of images acquired at different cardiac phases whereas T2 map did not show any difference in T2 values (p-value = 0.86).

### Animal Results

**Figure 6:**
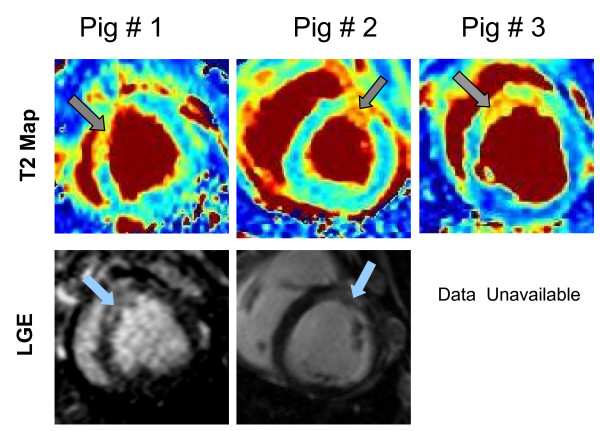
**Animal study results**. T2 maps from three pigs are shown in the top row and LGE images from two of the three pigs in the bottom row. Note that the LGE image for pig #2 was acquired in a slightly more basal slice position than the T2 map, but nevertheless demonstrates the expected myocardial infarction in the LAD territory. Pig # 3 died before the LGE image could be acquired, but 90 minutes of LAD occlusion virtually assures myocardial infarction.

The T2 maps in all animals showed a significantly higher T2 in the anterior segment associated with the occluded LAD segment compared with a remote inferior wall segment (for each animal, p-value < 0.001; one-sided t-test). In two pigs the necrotic tissue as shown by LGE matched the regions of T2 enhancement. The third animal died just before the injection of contrast agent and hence the LGE images were not available. Figure 6 and Table 6 summarize the results from the pig studies. While histological data was not available for these animals, 90 minutes of LAD occlusion is sufficient to cause infarction [24], as verified in two of the three animals by LGE.

**Table 6:** Results from the porcine model of acute myocardial infarction.

Pig #	Infarcted segment T2 (ms)	Remote segment T2 (ms)	p-value (one-sided)
1	83.6 ± 10	58.4 ± 7.2	< 0.001
2	83.5 ± 11	58.6 ± 4.5	< 0.001
3	80.4 ± 6.8	51.1 ± 5.6	< 0.001

In each animal, T2 in the infarcted segment was significantly higher (p-value < 0.001) than the T2 of the remote viable segment.

### Patient Results

**Figure 7:**
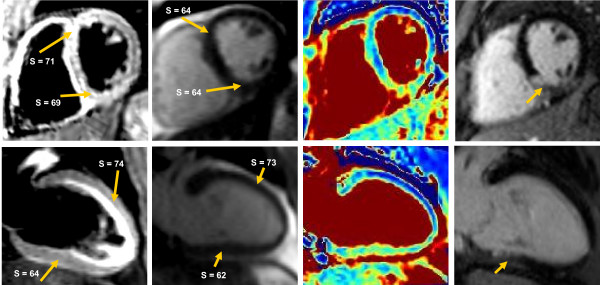
**SAX and VLA images from patient # 1**. Coronary angiography confirmed >90% occlusion of RCA. STIR images (1st column) did not show hyperintensity in inferior regions. In addition, the VLA images show high signal from static endocardial blood. Similarly, the infarcted region could not be detected in T2p-SSFP image with T2p = 55 ms (column 2). T2 maps (column 3) demonstrate increased T2 in the inferior regions (see Table 5) and the endocardial borders are clearly demarcated. LGE images (column 4) show the location and extent of the infarct. **S **= Signal Intensity.

Figure 7 shows SAX (top row) and VLA (bottom row) views generated using triple-inversion STIR (column 1), T2p-SSFP with TE_T2P _= 55 ms (column 2), T2 maps (column 3) and LGE (column 4) from patient #1. The quantitative results for all three patients are listed in Table 7. The T2 measured in the basal inferior regions in both views was significantly higher than the remote (basal anterior) region. This increased T2 in inferior region was undetectable in the STIR image, its contrast possibly compromised by the surface coil sensitivity inhomogeneity. Note also in the STIR VLA images the high signal from stagnant blood pool that makes it difficult to mark the endocardial border. These artifacts were not an issue in T2 maps. The LGE images confirm the localization of infarct.

**Table 7:** Summary of results from patients.

Patient #	Region of infarction	T2 (ms)		T2W (signal intensity)	
		
		Infarct	Remote	Infarct	Remote
1	Basal inferior	65.7 ± 6.7	46.5 ± 5.5	69 ± 8.6	71 ± 5.5

2	Mid inferior	68.9 ± 3.6	53 ± 3.2	100 ± 6.9	66.3 ± 8.3

3	Mid anterior/anteroseptal/inferoseptal	65.4 ± 2.3	52.1 ± 7	108.3 ± 9.6	48 ± 7.9

T2 measurements were made in a single mid-ventricular short-axis slice.

## Discussion

The sensitivity of T2W imaging to myocardial edema was first reported by Higgins [1] over 25 years ago, and several improvements on the original gated T2W spin echo sequence have been made since that time [10,14,15,25]. However, a variety of technical limitations including sensitivity to myocardial motion, surface coil intensity variation, high sub-endocardial signal from static blood, and the subjective nature of image interpretation have restricted the ability to confidently discern true myocardial T2 hyper-intensity with T2W imaging. This study demonstrated a rapid and practical technique for quantitative T2 mapping that addresses these limitations associated with conventional T2W imaging, and offers a potentially more reliable method for detection of myocardial edema.

### Motion Sensitivity

The sensitivity of myocardial signal to motion has been an inherent limitation of techniques such as T2W TSE and STIR imaging where cardiac motion can result in reduced myocardial signal (Figure 5); this problem is often observed in the highly mobile basal short axis plane [26]. The T2 preparation used in this work is non-selective, rendering this technique less sensitive to myocardial signal wash-out than turbo spin echo based methods. This was shown in normal volunteers where T2 values were unaffected by the location of the acquisition window in cardiac cycle, while myocardial signal in T2W STIR was significantly affected. Additionally, the single-shot SSFP readout is much less sensitive to artifacts induced by cardiac arrhythmias or imperfect breath-hold than segmented k-space methods.

### Surface Coil Intensity Variation

Variability in myocardial signal in T2W imaging can also result from surface coil intensity inhomogeneity, another important factor limiting the applicability of myocardial edema imaging. Results obtained under conditions of severe surface coil signal inhomogeneity showed that T2 mapping reduced the mean coefficient of variation among myocardial segments from 27.7% with T2W STIR, to 4.25% in the T2 maps (Figures 4A and 4B); the same level of variation is observed in T2 maps acquired using combined anterior and posterior elements. Attempts have been made [7,27] to compensate for surface coil inhomogeneities by using a large, single-element body-coil receiver; however, this approach comes with a SNR penalty [11] and an increase in scan time as parallel acquisition techniques cannot be used. Alternatively, coil intensity correction algorithms have been proposed [14,15] but have not yet been evaluated in clinical studies. T2 mapping solves this critical problem that will gain significance as the number of array coil elements increases to facilitate faster imaging.

### Stagnant Blood

Another significant problem recognized even in the first papers on in-vivo T2W imaging of the human heart [28,29] is sub-endocardial bright signal artifact caused by stagnant blood. Direct quantification of T2 easily distinguishes blood from myocardium [29], within the spatial resolution limits of the sequence. While a formal study of this artifact was not possible, we noted in the patient images the presence of high signal in endocardial blood (Figure 6). This was not observed in the T2 maps, although as mentioned earlier, partial volume effects in short-axis apical slices can artificially elevate the T2 in those segments.

### Advantages of Quantification

Yet another advantage of T2 mapping over T2W imaging is that it is a quantitative method. Most clinical studies using T2W imaging to date have relied on subjective visual assessment to detect regions of relative T2 signal hyperintensity [6,7]. Such an approach is purely qualitative and image interpretation can be challenging, particularly in the presence of the aforementioned artifacts and signal variability. The broad range of historical T2 values (Table 1) suggests that T2 estimation depends on methodology; however, defining the expected range of myocardial T2 values in normal subjects for a given technique enables it to be used to quantitatively asses abnormalities [9]. While our preliminary results in a small sample population did indicate significant inter-subject myocardial T2 variability, this was not correlated with heart rate, indicating that variable relaxation of magnetization between acquisitions was not the cause. Indeed, the phantom study demonstrated the robustness of the protocol over a range of wait times between TE_T2P _images (two through five RR intervals). There is evidence that myocardial T2 may vary depending on the recent health status of the individual. A recent study in high-endurance athletes suffering from common cold virus [30] showed evidence of myocardial edema and inflammation, both of which increased at four week follow-up. Although the normal subjects that participated in our study were healthy and free from any history of cardiovascular disease, we did not record information regarding ongoing or recent medical conditions such as recent infections. In addition, studies on skeletal muscle [31,32] have suggested activity-dependent changes in T2. Although similar changes have not been demonstrated in myocardium, the possibility of this effect cannot be ruled out.

Quantification of T2 offers a distinct advantage over T2W imaging in the detection of global changes in myocardium as may be expected to occur in myocarditis [27] or cardiac allograft rejection [9]. A recent published white paper [8] recommends the use of a CMR protocol that includes T2W imaging as part of a comprehensive diagnostic approach in patients with suspected myocarditis. In such cases, T2W techniques that rely on regional differences in myocardial signal may fail, whereas T2 mapping will facilitate detection of global or diffuse changes in myocardium. Additionally, T2 mapping will enable accurate monitoring of the treatment and/or progression of disease.

## Limitations

While our human myocardial T2 results concurred with previously published work (Table 1), phantom results showed an overestimation of T2 values; we identified two probable causes of this inaccuracy.

First, the k-space ordering chosen for SSFP readout was linear instead of centric despite the higher T2 accuracy in phantoms provided by the latter. However, as shown in Figure 2, centric ordering gave rise to artifacts in the *in vivo *images, possibly due to the oscillatory approach to steady-state having a stronger effect on the central k-space lines. Images and T2 maps acquired using linear ordering did not show this artifact and demonstrated significantly less spatial variation in calculated T2. The overestimation in T2 with linear ordering was likely caused by the acquisition of central k-space lines after the T2p magnetization has been perturbed by a number of RF excitation pulses and the signal has begun to approach SSFP steady-state.

A second probable cause of T2 overestimation may be the number of echo times and fitting method used. Given that our mono-exponential model has two parameters (M_o _and T2) we chose to use three images for the T2 calculation as the minimum required for a least-squares linear-regression fitting of the logarithmically transformed data to minimize breath-hold times. A recent work by Sparrow et al. [33] demonstrated the feasibility of this approach. The effect of noise and its propagation into the T2 map through the linear least-squares fitting process has not been analyzed in this study, but the procedure would be similar to the one described by MacFall et al. [34]. While the use of additional echoes may provide robustness to a non-linear fit, it would increase the breath-hold time and patient discomfort. The single shot SSFP readout and use of only three TE_T2P _times ensures the breath-hold time (7RR) is within the capabilities of most of the patients.

There is still the potential for mis-registration between images causing error in the T2 maps. If breath-holding is not feasible, the sequence could be combined with respiratory triggering and/or image registration methods could be applied prior to T2-map calculation. Although a recently described T2W imaging technique [15] used a motion correction algorithm prior to averaging to enhance SNR, this was accomplished using rigid-body registration of multiple images of identical contrast. In this study the T2p times, and hence the contrasts, of the three images were different; consequently it is unknown how this or other motion correction algorithms would perform.

The analyses of T2 variability between myocardial segments showed no statistically significant differences among the twelve basal and mid-ventricular segments; however, our results suggest a consistent overestimation of T2 in the four apical segments (Figure 3). One potential reason for this effect could be the inclusion of cavity blood in apical segments due to partial volume effects caused by the tissue geometry, although this theory was not explored in detail. Excluding the apical sectors reduced the mean spatial T2 variation from 5.3% to 3.8%. To reduce partial volume effects, one may either use thinner slices, or HLA and VLA views to measure T2 in the apical segments. The feasibility of this second approach is supported by the results showing that T2 maps from mid-ventricular short-axis and long-axis views demonstrated no significant difference.

## Conclusions

Detection of myocardial edema is not possible by other cardiac imaging modalities, and the lack of a robust CMR approach has limited broad application of this potentially important measure of myocardial status. This work has demonstrated an accurate, fast, quantitative approach to detect the elevated T2 associated with myocardial edema that successfully addresses the well-known limitations of T2W imaging. Further studies are warranted to evaluate the clinical applicability of this technique in the range of conditions that are known to globally or regionally affect myocardial water content.

## Competing interests

YCC is an employee of Siemens Healthcare. OPS and SVR receive research grant support from Siemens Healthcare.

## Authors' contributions

SG, SVR and OPS planned and designed the study. SG, OPS, and YCC designed and implemented the T2-mapping technique. GM constructed the phantoms and measured their relaxation parameters. AM recruited patients and acquired patient data. Data was analyzed by SG. All authors participated in manuscript drafting and editing and approved the final version of the submitted manuscript.
